# Pharmacogenetic association with early response to intravitreal ranibizumab for age-related macular degeneration in a Korean population

**Published:** 2013-03-21

**Authors:** Woohyok Chang, Dong Hyoun Noh, Min Sagong, In Taek Kim

**Affiliations:** 1 Department of Ophthalmology, Yeungnam University College of Medicine, Daegu, South Korea; 2 Department of Ophthalmology, School of Medicine, Kyungpook National University, Daegu, South Korea

## Abstract

**Purpose:**

To determine whether genetic factors that influence age-related macular degeneration (AMD) have an early pharmacogenetic effect on treating exudative AMD with ranibizumab in a Korean population.

**Methods:**

A retrospective study of 102 patients (70 with typical neovascular AMD and 32 with polypoidal choroidal vasculopathy) with exudative AMD treated with intravitreal ranibizumab monotherapy was conducted. Optical coherence tomography, fluorescein, and indocyanine green angiography were taken at the baseline. The best-corrected visual acuity (BCVA) and the central subfield macular thickness (CSMT) were recorded at the baseline and at each monthly visit. The genotypes of the polymorphisms in the known AMD susceptibility loci (*CFH, AMRS2, HTRA1, VEGFA, and KDR*) were determined, and association between their frequencies and the changes in the BCVA and the CSMT were evaluated.

**Results:**

The mean baseline visual acuity was 0.96±0.59 logMAR (approximately 20/200 in the Snellen equivalent), and the mean number of injections was 3.87 before the month 6 visit. No association was observed between the change in BCVA and each genotype. For the changes in the CSMT, a significant difference was observed only with the *VEGF-A* (rs833069) gene. The decrease in the CSMT at month 3 for the major allele homozygote AA genotype, the heterozygote AG genotype, and the risk allele homozygote GG genotype was 25.66±85.40, 86.93±92.31, and 85.30±105.30 μm, respectively (p=0.012, p=0.044, and p=0.002 for AG, GG, and combined AG or GG genotype, respectively, compared to the AA genotype). This trend was maintained until month 6.

**Conclusions:**

The *VEGF-A* (rs833069) polymorphism showed a significant association with the anatomic response to intravitreal ranibizumab. No significant difference was found between the genotype of the potential risk polymorphism for development of AMD and the early visual improvement after intravitreal ranibizumab.

## Introduction

Age-related macular degeneration (AMD) is a leading cause of irreversible visual impairment estimated to affect more than 50 million people worldwide [1]. Patients in advanced stages of AMD develop choroidal neovascularization (CNV) or atrophic changes in the central macula. As a result, patients have low vision, which eventually leads to legal blindness. The major risk factors for AMD include increasing age and smoking. Although the mechanism for AMD is unclear, several established factors are known to be involved in angiogenesis. Vascular endothelial growth factor (VEGF) appears to be the most important among them.

Ranibizumab (Lucentis; Genentech, Inc., South San Francisco, CA) is an anti-VEGF agent that targets all VEGF-A isoforms and their biologically active degradation products [2]. Intravitreal anti-VEGF injection is currently the standard treatment for exudative AMD. Anatomically, ranibizumab treatment is associated with arrested CNV growth and reduced leakage from CNV. Retinal thickness on optical coherence tomography (OCT) was reduced dramatically after initial treatment, and this reduction was associated with mean improvement in visual acuity [3,4]. Through several years of clinical experience of anti-VEGF, we recognized that there is a broad spectrum of clinical response, with a substantial proportion of patients experiencing further visual deterioration in spite of ongoing treatment [5].

Genetic factors also play a substantial role in the etiology of AMD and its overall severity [6]. Recently, a thymine-to-cytosine (T-to-C) transition in the complement factor H gene (*CFH*, Y402H) was found to be strongly associated with AMD [7-9]. Through family linkage studies and fine mapping, another two major nearby genes associated with AMD were identified: age-related maculopathy susceptibility 2 (*ARMS2*) and high temperature requirement factor A1 (*HTRA1*) [10-12].

VEGF is involved in angiogenesis for exudative AMD and has been a target for treating exudative AMD. VEGF acts through specific tyrosine kinase receptors, of which VEGF receptor-2 (VEGFR-2) is a high-affinity receptor for VEGF-A, and mediates most of the endothelial growth and survival signals from VEGF-A. An association between a polymorphism in *VEGF* and its receptor gene with the development of AMD has also been reported [13,14]. Galan et al. [14] reported that two polymorphisms (rs833069 in intron 2 of the *VEGF-A* gene, rs2071559 in the promoter of the kinase insert domain receptor [*KDR*] gene) were significantly associated with the development of AMD. In particular, for *VEGF-A* rs833069 the AMD risk was increased fivefold for G homozygotes compared with the homozygous carriage of the A allele. For *KDR* rs2071559, the AMD risk was increased threefold for T homozygotes compared with the homozygous carriage of the C allele.

Recently, several pharmacogenetic studies among Caucasian populations reported a relationship between genetic characteristics and response to anti-VEGF treatment. However, the phenotypic and genotypic characteristics of Asian AMD are unique from those of Caucasian AMD including the proportion of polypoidal choroidal vasculopathy (PCV) and frequency of *CFH* Y402H [15-17]. Therefore, identifying the genetic associations in Asian populations that may predict the response to ranibizumab, the current standard treatment for exudative AMD, is important. Accordingly, this study evaluated the association of the genotype for the *CFH*, *ARMS2*, *HTRA1*, *VEGF-A*, and *KDR* genes with the change in visual acuity and macular thickness after 6 months of intravitreal ranibizumab therapy for exudative AMD in a Korean population.

## Methods

### Patients

This study was approved by the Institutional Review Board of Yeungnam University Medical Center. All subjects provided written informed consent before participation. The research adhered to the tenets of the Declaration of Helsinki. All individuals were recruited from the Department of Ophthalmology, Yeungnam University Medical Center, and underwent a clinical examination by two retina specialists.

All patients were examined with best-corrected visual acuity (BCVA), fundus photography, fluorescein, indocyanine green angiography, and optical coherence tomography (Stratus OCT; Carl Zeiss, Jena, Germany). The BCVA was measured at the initial presentation and at each follow-up visit. The BCVA was converted to the logarithm of minimum angular resolution (logMAR) values for the calculation. Fluorescein and indocyanine green angiography (HRA2, Heidelberg Engineering, Heidelberg, Germany) were performed at the initial presentation for all patients. PCV was diagnosed primarily based on the indocyanine green angiographic findings, branching vascular network and terminating polypoidal lesion(s). The central subfield macular thickness (CSMT) was measured with the fast macular thickness map protocol at the baseline and at each follow-up visit. The inclusion criteria were age 60 years or more, diagnosis of exudative AMD in one or both eyes, and minimum 6 months of monthly follow-up after the first intravitreal ranibizumab injection. Eyes with subfoveal atrophy, CNV secondary to pathologic myopia, angioid streak, and a previous history of photodynamic therapy or anti-VEGF injection were excluded. We included the second eye only if both eyes met the inclusion criteria. All patients underwent three consecutive intravitreal ranibizumab injections in the loading phase and further injections as required in the maintenance phase. Retreatment was prompted only if signs of lesion activity (i.e., persistent or recurrent subretinal fluid, intraretinal cysts or thickening on OCT, or new subretinal hemorrhage on fundus examination).

### Genotype determination

Genomic DNA was extracted from a buccal swab using a Qiagen QIAamp Mini Kit (Qiagen, Valencia, CA), and the DNA concentration was determined using a NanoDrop ND1000 (Wilmington, DE) spectrophotometer. The purity of the DNA was assessed based on the 260/280 nm absorbance ratio ranging from 1.7 to 2.1. Genotyping was undertaken using the Sequenom (San Diego, CA) iPLEX platform, according to the manufacturer's instructions. Five single nucleotide polymorphisms (SNPs; rs1061170, rs10490924, rs11200638, rs833069, and rs2071559) were detected by analyzing the primer extension products generated from previously amplified genomic DNA using a Sequenom chip-based matrix-assisted laser desorption/ionization time-of-flight (MALDI-TOF) mass spectrometry platform. Multiplex SNP assays were designed using SpectroDesigner software (Sequenom). Polymerase chain reaction (PCR) amplification took place in a 5 µl mixture containing 10 ng of genomic DNA, 100 nM of each amplification primer, a 500 mM dNTP mix, 1.625 mM MgCl_2_, and 5.5 units HotStarTaq DNA Polymerase (Qiagen). The mixture was subjected to the following PCR conditions: a single denaturation cycle at 95 °C for 15 min, followed by 45 cycles at 94 °C for 20 s, 56 °C for 30 s, 72 °C for 60 s, and a final extension at 72 °C for 3 min. The unincorporated nucleotides in the PCR product were deactivated using shrimp alkaline phosphatase. The allele discrimination reactions were conducted by adding the allele-specific extension primers (UEP), DNA polymerase, and a cocktail mixture of deoxynucleotide triphosphates and dideoxynucleotide triphosphates to each well. MassExtend clean resin (Sequenom Inc.) was added to the mixture to remove the extraneous salts that could interfere with MALDI-TOF analysis. The primer extension products were then cleaned and spotted onto a SpectroChip (Sequenom Inc.). The genotypes were determined by spotting an aliquot of each sample on to a 384 SpectroChip, which was then read with the MALDI-TOF mass spectrometer. Table 1 lists the primer sequences.

**Table 1:** Sequenom MassARRAY Prime sequences

**Gene** **SNP Number**	**Region**	**Primer Name**	**Sequence (5’ to 3’)**
*CFH* rs1061170	Y402H	Forward Reverse UEP	ACGTTGGATGGTTATGGTCCTTAGGAAAATGACGTTGGATGACGTCTATAGATTTACCCTGCTGTACAAACTTTCTTCCAT
*ARMS2* rs10490924	A69S	Forward Reverse UEP	ACGTTGGATGAAGCAGAGGAGCAAACTGTCACGTTGGATGAAGAAGGCTGGTAAGCAGAGAGCTCAGTGTGGATTTTAG
*HTRA1* rs11200638	Promoter	Forward Reverse UEP	ACGTTGGATGTCACGCGCTGGTTCTGCCCACGTTGGATGTTTCTGCCAGCTCCGCGGACGGACGCTGCCTTCGTCC
*VEGF-A* rs833069	Intron	Forward Reverse UEP	ACGTTGGATGTGTGACCCTTCCTGTGTGAGACGTTGGATGCCCAAAGGAATGCAAACCAGTCAGCCTAATGGGATCTC
*KDR* rs2071559	Promoter	Forward Reverse UEP	ACGTTGGATGATCAGAAAACGCACTTGCCCACGTTGGATGGGTCACTTCAAACTTGGAGCGGGAAATAGCGGGAATG

SNP=single nucleotide polymorphism; UEP=unique-event polymorphism

### Data analysis

An independent Student *t* test was conducted to determine the statistical significance of the potential predictor variables. The association between the genotype and the change in the BCVA and the CSMT was assessed with one-way analysis of the variance. The comparisons between the genotypes were adjusted for multiple comparisons using Tukey’s multiple comparison test. The predetermined level of statistical significance was p<0.05. All analyses were performed using commercially available software (SPSS v18.0K; SPSS Inc., Chicago, IL).

## Results

This study evaluated 102 patients with AMD treated with ranibizumab across the five known genetic risk factors for AMD. Table 2 lists the baseline demographics, lesion subtype, BCVA, and the CSMT. The mean baseline visual acuity was 0.96±0.59 logMAR (approximately 20/200 in the Snellen equivalent), and the mean number of injections was 3.87 before the month 6 visit. The association of smoking status, lesion subtype, and baseline BCVA with the change in the BCVA and the CSMT at 6 months was analyzed (Table 3 and Table 4). Table 5 and Table 6 show the changes in BCVA and CSMT from the baseline according to the genotypes examined at 3 and 6 months after the intravitreal ranibizumab injection, respectively. There was no significant statistical difference between change in BCVA and each genotype. For the change in the CSMT, however, a significant difference was observed for the *VEGF-A* gene. The decrease in the CSMT at month 3 for the major allele homozygote AA genotype, the heterozygote AG genotype, and the risk allele homozygote GG genotype was 25.66±85.40, 86.93±92.31, and 85.30±105.30 μm, respectively (p=0.012, p=0.44, and p=0.002 for AG, GG, and combined AG or GG genotype compared with the AA genotype). This trend was also observed at month 6 (Table 7). No association was observed between the CSMT changes and the genotype for the *CFH*, *ARMS2*, *HTRA1*, and *KDR* genes.

**Table 2:** Demographic features of the patients at the baseline

Characteristics	Values
Number of patients	102
Age (mean±SD)	69.12±8.82
Gender (male/female)	67/35
Subtype	
-Typical nAMD	70(68.6%)
- PCV	32(31.4%)
Central subfield macular thickness, µm (mean±SD)	
-Typical nAMD	282.34±78.47
-PCV	293.38±84.37
LogMAR vision (mean±SD)	0.96±0.59
Mean BCVA (approximate Snellen equivalent)	20/200

BCVA=best-corrected visual acuity; LogMAR=logarithm of minimum angular resolution; nAMD=neovascular age-related macular degeneration; PCV=polypoidal choroidal vasculopathy; SD=standard deviation

**Table 3:** Association of the smoking status, lesion subtype and baseline BCVA with BCVA at month 6

**Potential variables**				**p values**
**Smoking status**	**Never smoked**	**Ex-smokers**	**Current smokers**	
Number of cases	41	17	44	
Mean baseline BCVA	0.99±0.53	0.70±0.62	0.97±0.61	0.19/ 0.96
Mean BCVA improvement	0.06±0.37	0.15±0.31	0.07±0.36	0.63/ 0.80
**Lesion subtype**	**Typical nAMD**	**PCV**		
Number of cases	70	32		
Mean baseline BCVA	0.90±0.57	1.00±0.63		0.44
Mean BCVA improvement	0.10±0.35	0.00±0.32		0.42
**Baseline visual acuity**	**<20/100**	**20≥100**		
Number of cases	55	47		
Mean baseline BCVA	1.05±0.62	0.80±0.53		0.04
Mean BCVA improvement	0.11±0.37	0.04±0.34		0.28

Never smoked is the reference category (one-way ANOVA). BCVA=best-corrected visual acuity, LogMAR vision; nAMD=neovascular age-related macular degeneration; PCV=polypoidal choroidal vasculopathy

**Table 4:** Association of the smoking status, lesion subtype and baseline BCVA with CSMT change at month 6

**Potential variables**				**p values**
**Smoking status**	**Never smoked**	**Ex-smokers**	**Current smokers**	
Number of cases	41	17	44	
Mean baseline CSMT	268.27±64.85	293.35±76.82	299.23±93.68	0.53/ 0.18
Mean CSMT decrease	39.07±69.27	93.88±71.76	71.66±103.91	0.08/0.20
**Lesion characteristics**	**Typical nAMD**	**PCV**		
Number of cases	70	32		
Mean baseline CSMT	282.34±79.04	293.38±85.71		0.526
Mean CSMT decrease	50.53±92.29	80.06±105.10		0.051
**Baseline visual acuity**	**<20/100**	**20≥100**		
Number of cases	55	47		
Mean baseline CSMT	292.39±78.87	278.10±83.47		0.38
Mean CSMT decrease	66.04±81.29	57.85±95.90		0.64

Never smoked is the reference category (one-way ANOVA). CSMT=central subfield macular thickness (µm); nAMD=neovascular age-related macular degeneration; PCV=polypoidal choroidal vasculopathy

**Table 5:** Best-corrected visual acuity changes from the baseline for each genotype

dbSNP ID	Gene	logMAR Vision Change at month 3	P value	logMAR Vision Change at month 6	P value
rs1061170	CFH	0.0817(83)/0.0465(17)/-0.1505(2)	0.629	0.0835(83)/0.0641(17)/-0.0880(2)	0.791
rs10490924	ARMS2	0.0689(8) /0.0471(37)/0.0873(57)	0.867	0.1285(8) /0.1010(37)/0.0540(57)	0.756
rs11200638	HTRA1	0.0947(9) /0.0342(37)/0.0920(56)	0.731	0.1672(9) /0.0833(37)/0.0581(56)	0.694
rs833069	VEGF-A	0.0157(38)/0.0963(41)/0.1704(23)	0.115	0.0542(38)/0.0757(41)/0.1166(23)	0.807
rs2071559	KDR	0.0762(43)/0.0449(47)/0.1472(12)	0.618	0.0620(43)/0.0679(47)/0.1652(12)	0.662

CFH=complement factor H; ARMS2=age related maculopathy susceptibility protein 2; HTRA1=high-temperature requirement A-1; logMAR=logarithm of minimum resolution; SNP=single nucleotide polymorphism; VEGF=vascular endothelial growth factor; KDR=kinase insert domain receptor. Data are mean logMAR vision change from baseline (n) for each genotype; major allele homozygous/heterozygous/risk allele homozygous.

**Table 6:** Change in the central subfield macular thickness from the baseline for each genotype

dbSNP ID	Gene	CSMT Change at month 3	P value	CSMT Change at month 6	P value
rs1061170	CFH	66.07(83)/54.76(17)/43.00(2)	0.868	63.77(83)/56.35(17)/50.00(2)	0.934
rs10490924	ARMS2	39.00(8)/57.65(37)/71.16(57)	0.609	54.88(9) /63.59(37)/62.44(57)	0.969
rs11200638	HTRA1	52.67(9)/53.54(37)/72.25(56)	0.622	69.67(9)/58.32(37)/63.68(56)	0.928
rs833069	VEGF-A	25.66(38)/86.93(41)/85.30(23)	0.008	28.71(38)/83.66(41)/79.57(23)	0.011
rs2071559	KDR	43.42(43)/76.23(47)/87.58(12)	0.182	55.14(43)/64.51(47)/79.00(12)	0.692

CFH=complement factor H; ARMS2=age related maculopathy susceptibility protein 2; HTRA1=high-temperature requirement A-1; CSMT=central subfield macular thickness; SNP=single nucleotide polymorphism; VEGF=vascular endothelial growth factor; KDR=kinase insert domain receptor. Data are mean logMAR vision change from baseline (n) for each genotype; major allele homozygous/heterozygous/risk allele homozygous.

**Table 7:** Association between *VEGF-A* rs833069 polymorphism and a change in central subfield macular thickness after the ranibizumab treatment

**dbSNP ID**	**CSMT Change at month 3**	**P value**	**CSMT Change at month 6**	**P value**
*VEGF-A* rs833069	AA : GA AA : GG AA : GA, GG	0.012 0.044 0.002	AA : GA AA : GG AA : GA, GG	0.014 0.065 0.001

VEGF=vascular endothelial growth factor; SNP=single nucleotide polymorphism; CSMT=central subfield macular thickness

## Discussion

In this study, we found a significant association between *VEGF-A* (rs833069) genotype variants and anatomic response to intravitreal ranibizumab in patients with AMD. However, there was no association in visual improvement for any of the genes studied, *CFH* (rs1061170), *ARMS2* (rs10490924), *HTRA1* (rs11200638), *VEGF-A* (rs833069), and *KDR* (rs2071559).

Previous studies have investigated a possible association between the *CFH* genotype and anti-VEGF treatment response, mostly in Caucasian populations. With intravitreal bevacizumab, Brantley et al. [18] and Nischler et al. [19] reported a trend toward worse visual outcome and a lower chance of improving visual acuity for the CC genotype. With intravitreal ranibizumab, Lee et al. [20] reported that the *CFH* genotype did not affect the post-treatment visual outcome, but the CC genotype was more likely to require more injection than the TT genotype. However, Francis [21] and McKibbin et al. [22] reported different results showing that the CC genotype required fewer injections and had a more favorable visual outcome. Menghini et al. [23] recently reported that in a long-term study the CT genotype had an approximately three times higher probability of experiencing a significant long-term gain in visual acuity at 12 and 24 month follow-up. In addition to these confusing results, another study of Caucasians by Orlin et al. [24] reported no association between the *CFH* genotype and the response to anti-VEGF. In our study, there was no significant visual outcome difference between the TT genotype and the CT genotype.

*ARMS2/HTRA1* is the second major polymorphism associated with AMD which is localized in  chromosome 10q26. Brantley et al. [18] and Orlin et al. [24] reported no association of the anti-VEGF treatment outcome with an *ARMS2* polymorphism, but McKibbin et al. [22] observed a better visual outcome with the risk allele G in the *HTRA1* gene through a +2.2 and +7.5 EDTDRS letter score change in the GG and GA genotypes, respectively. Unlike the *CFH* gene, the incidence of the *ARMS2/HTRA1* polymorphism appears to be similar in Caucasians and Asians, and this polymorphism is strongly associated with PCV and AMD [15,16,25-27]. A high incidence of polymorphism was observed in the present study population, the number of homozygotes of each risk allele was more than half, and there was no significant association with the response to ranibizumab.

The *VEGF* gene does not appear to be a major genetic risk factor for developing exudative AMD. Considering the role of VEGF in angiogenesis and hyperpermeability (the major characteristics of exudative AMD), the polymorphism in the *VEGF* and *VEGF* receptor gene might be a factor that affects the pharmacological mechanism of anti-VEGF. With this theoretical base, we selected two SNPs, *VEGF-A* (rs833069) and *KDR* (rs2071559), which Galan et al. recently reported were associated with the development of AMD [14]. Recently, Park et al. [26] reported that an rs833069 polymorphism in *VEGF-A* was significantly associated with the risk of PCV in a Korean population. They revealed a 2.29-fold increased risk in the risk allele G compared to the major allele A and a 6.25-fold increased risk of PCV in the GG genotype compared to the major allele AA genotype. Several studies reported an association of the *VEGF* gene with the treatment response. McKibbin et al. [22] reported an association between a variation of the *VEGF-A* gene (rs1413711) and the response to ranibizumab, and Nakata et al. [28] observed a better visual outcome with the *VEGF-A* rs699946 GG genotype after intravitreal bevacizumab in a Japanese population. These findings suggest that many components of the VEGF system are involved in the treatment outcome. In this Korean population–based study, the risk allele G of rs833069 was significantly associated with a better anatomic outcome and had a tendency to be associated with improvement in the mean BCVA after ranibizumab loading, but the improvement was not statistically significant. Most prior studies did not use the CSMT as an outcome measure. The visual outcome is an integrated outcome that reflects the functional ability of the visual system, but is still a subjective measure for assessing the pharmacogenetic response, particularly in a retrospective study. Moreover, the CSMT, particularly measured at month 3, is relatively objective because the CSMT data measured with OCT are not biased by drug dosage and ocular conditions other than AMD.

The marker rs833069 is located in intron 2 of the *VEGF-A* gene. There are no functional data on this polymorphism. However, the *VEGF-A* rs833069 polymorphism has been reported to be associated with the development of AMD in Caucasians [14] and PCV in Asians [26]. Lima et al. [29] reported that PCV might be a subset of AMD with similar demographic and genetic risk factors and clinical features through a Caucasian patient–based genetic study. In the present study, there was a significant association between the rs833069 polymorphism and the change in CSMT in the heterogeneous patient group, which includes typical neovascular AMD and PCV.

With recent advances in OCT technology, studies of exudative AMD have recently reported that foveal thickness measurements were useful in evaluating treatment response to intravitreal ranibizumab [30,31]. OCT is extremely useful in detecting intraretinal and subretinal fluid and is thus important for evaluating the treatment response. However, the relationships between visual acuity and central retinal thickness were inconsistent. According to a recent report [32], improvement in the inner segment/outer segment photoreceptor junction (IS/OS) line after anti-VEGF injection would be a good indicator for predicting the initial response to anti-VEGF treatment. However, the present study based on stratus OCT was limited in analyzing the IS/OS line in detail, and thus, we used the CSMT as the only OCT parameter, which might cause the discrepancy between visual and anatomic outcomes in the *VEGF-A* rs833069 polymorphism.

To our knowledge, no other group has investigated the influence of the genotype on the response to ranibizumab therapy in an Asian population, and the CSMT was used as a secondary parameter to assess the treatment outcome to overcome the limitation of a retrospective study when evaluating the pharmacogenetic response. The change in the CSMT should be considered another good indicator of the treatment response while eliminating subjective bias, particularly the interval change after the loading phase. However, this study is limited by small sample size, relative low minor allele frequency in some SNPs, and short follow-up period.

In conclusion, this study evaluated the potential association between the selected SNPs in the *CFH*, *ARMS2*, *HTRA1*, *VEGF-A*, and *KDR* genes, and the response to an intravitreal ranibizumab injection for exudative AMD in a Korean population. A significant association between the *VEGF-A* (rs833069) genotype variants and the anatomic response to intravitreal ranibizumab was observed, but no significant difference was found between the genotype of the potential risk polymorphism for the development of AMD and the visual acuity change after treatment with intravitreal ranibizumab. A larger cohort study including more potential risk SNPs for the development of AMD is needed to evaluate the pharmacogenetic association with the response to an anti-VEGF treatment.
